# Needs assessment for a curriculum for difficult conversations -a survey from 5 Chinese accredited neurology residency training programs

**DOI:** 10.1186/s12909-020-02246-7

**Published:** 2020-09-29

**Authors:** Lixin Zhou, Bo Wu, Liyong Wu, Xin Cheng, Bo Hu, Ming Yao, Yicheng Zhu, Bin Peng, Liying Cui, Jun Ni

**Affiliations:** 1 Department of Neurology, Peking Union Medical College Hospital (PUMCH), Chinese Academy of Medical Science (CAMS) and Peking Union Medical College (PUMC), Beijing, 100730 China; 2 Department of Neurology, West China Hospital, Sichuan University, Chengdu, Sichuan China; 3 Department of Neurology, Xuanwu Hospital Capital Medical University, Beijing, China; 4 Department of Neurology, Huashan Hospital Fudan University, Shanghai, China; 5 Department of Neurology, Union Hospital, Tongji Medical College, Huazhong University of Science and Technology, Wuhan, China

**Keywords:** Neurology residency training, Communication skill, Difficult conversation, Curriculum develop

## Abstract

**Background:**

Communication skill is a core competency in neurology residency training. Specific training in this area at the residency level is often lacking, especially regarding difficult conversations. The aim of this study is to evaluate the current state in teaching residents about difficult conversations in 5 Chinese accredited neurology residency training programs and determine whether there is a perceived need for a formalized curriculum in this field.

**Methods:**

An anonymous, 27-question, cross-sectional online survey addressing difficult conversations for neurological residents were distributed to five grade-A, class-3 hospitals selected from the affiliated teaching hospitals of medical schools qualified to provide neurology residency training in China.

**Results:**

A total of 182 residents responded to the survey, and the response rate was 67.16% (182/271). Of the participants, 84.6% were female and the average age was 26.8 years. The majority of respondent residents (*n* = 168; 92.31%) reported being exposed to at least one difficult conversation in their medical careers. Only 43 (23.63%) participants reported having previously received formal communication skills training. In comparison with residents without previous training, those with previous training indicated significantly more confidence (*P* = 0.003) and were under lower pressure (*P* = 0.037) in managing difficult conversations. Only 97 (53.3%) residents indicated interest in receiving formal training. Time, lack of enthusiasm, lack of educational materials and faculty expertise were commonly cited barriers to formalized training.

**Conclusion:**

This survey provides a preliminary assessment of the current status of education on the topic of difficult conversations in five accredited Chinese neurology residency training programs. Our results suggest that there is an unmet need to further develop and implement educational activities by teaching residents to lead difficult conversations. Future efforts should be made to establish and promote a standard and targeted communication curriculum in difficult conversation for Chinese neurological residents.

## Background

Communication skill is one of the core competencies in residency training and medical practice [1]. Neurologists regularly confront complex clinical scenarios that require reasonable decision-making and difficult communication with patients, families, and colleagues. Evidence has shown that effective communication could reduce adverse events and improve patients’ satisfaction and adherence to treatment [2, 3]. Previous studies have shown that the training of communication skills in under-graduate and post-graduate education is very limited in China. They pay more attention to theoretical knowledge, but ignore practical communication skills training [4]. Therefore, practicable and effective training on this topic is important at residency level. Unfortunately, specific curriculum on managing difficult conversations have not been described for Chinese neurology residents.

Under the guidance of the Ministry of Health, the Chinese Medical Association issued the mandatory resident training standards in 2012, and the government implemented a plan to carry out a three-year resident standardized training program nationwide from 2015 [5, 6]. By the end of 2014, 8500 residency training programs had been carried out in 559 hospitals, recruiting 55,000 residents [6, 7]. Although the national government has taken a big step forward in establishing training standards, these standards do not necessarily create high-quality resident training programs. Due to the vast territory of China, there are great diverse in medical care and education. In particular, doctor-patient communication is closely related to local culture and economy. Therefore, it is impossible to use a unified program to carry out resident communication skills training in China. We should develop individualized training programs according to the actual situation of various medical institutions and residency groups. Most of the large-scale teaching hospitals in China are located in the big cities with similar economic, medical and educational backgrounds, who are also the first hospitals to carry out standardized resident training program. Most patients in Neurology department of these hospitals are faced with challenges in diagnosis and treatment, and residents might encounter similar difficulties in doctor-patient communication. Therefore, we suppose that they may have consistent needs in communication skills training, and have the ability to carry out preliminary research on this topic in the future. Therefore, the objective of this study is to evaluate the current state in teaching residents about difficult conversations in 5 Chinese accredited neurology residency training programs and determine whether there is a perceived need for a formalized curriculum in this field.

## Methods

We developed an anonymous, cross-sectional online survey addressing difficult conversations for neurological residents. These questions overlapped across multiple resources, including the Accreditation Council for Graduate Medical Education (ACGME) guidelines for communication skills training in neurology residency, educational milestones for neurology residencies, and topics from a review of the literature [8–10]. The survey included Likert-style questions, multiple choice questions, and free-text boxes for qualitative responses. All questions were optional, and completion was not required to end the survey. The residents’ survey consisted of 27 questions (the full survey is available in Supplement 1). The original questionnaire is in Chinese. The supplemental material is a translated version. The questionnaire included the following areas: 1) basic demographic data, 2) resident’s knowledge, experience, and confidence regarding the management of difficult conversations, 3) previous formal training in communication skills during neurology residency, and 4) interest in receiving training on difficult conversations and barriers to implementation. The surveys were distributed and collected using the electronic online survey tool Wenjuanxing (www.wjx.cn, China).

Surveys were distributed to five accredited neurology residency programs in China. The five hospitals were all grade-A, class-3 teaching hospitals selected from the affiliated teaching hospitals of medical schools qualified to provide neurology residency training in China. An introductory letter that described the rationale and objective of the study was emailed to program directors for participation. Program directors were asked to distribute the survey to their residents to avoid direct contact between the authors and the residents. Informed consent was assumed if the respondent chose to complete the questionnaire.

### Statistical analysis

Statistical analyses were performed using SPSS version 19.0 for Windows (SPSS Inc.). All *p* values were two-tailed and criteria for significance were *p* < 0.05. Standard descriptive statistics were used. Comparison between residents with and without previous training were performed using analysis of variance followed by Fisher LSD post hoc tests.

## Results

From the number of residents in the five programs, it is assumed that the surveys were distributed to 271 residents. A total of 182 residents responded to the survey; therefore, the response rate was 67.16% (182/271). The highest proportion of respondents was from Peking Union Medical College Hospital (*n* = 49; 83.05%), followed by Xuanwu Hospital Capital Medical University (*n* = 65; 81.25%), West China Hospital Sichuan University (*n* = 34; 56.67%), Wuhan Union Hospital Huazhong University of Science and Technology (*n* = 21; 47.73%), and Huashan Hospital Fudan University (*n* = 13; 46.43%).

### Residents’ demographic characteristics

The demographic features of the 182 resident respondents are listed in Table 1. Of the participants, 84.6% were female. The average age was 26.8 years. Residents from all postgraduate year (PGY) levels, from PGY1 to PGY5, completed the survey.

**Table 1:** Residents’ demographic characteristics

	Resident	%
Age Range		
20–29	155	85.16
30–39	26	14.29
40–49	1	0.55
Gender		
Female	154	84.62
Male	28	15.38
Postgraduate level		
PGY1	65	35.71
PGY2	55	30.22
PGY3	45	24.73
PGY4	6	3.3
PGY5	11	6.04
Hospital and University		
Peking Union Medical College Hospital	49	26.92
Xuanwu Hospital Capital Medical University	65	35.71
West China Hospital Sichuan University	34	18.68
Union Hospital, Tongji Medical College, Huazhong University of Science and Technology	21	11.54
Huashan Hospital Fudan University	13	7.14

### Residents’ knowledge, experience, and confidence regarding difficult conversations in neurology

**Fig. 1:**
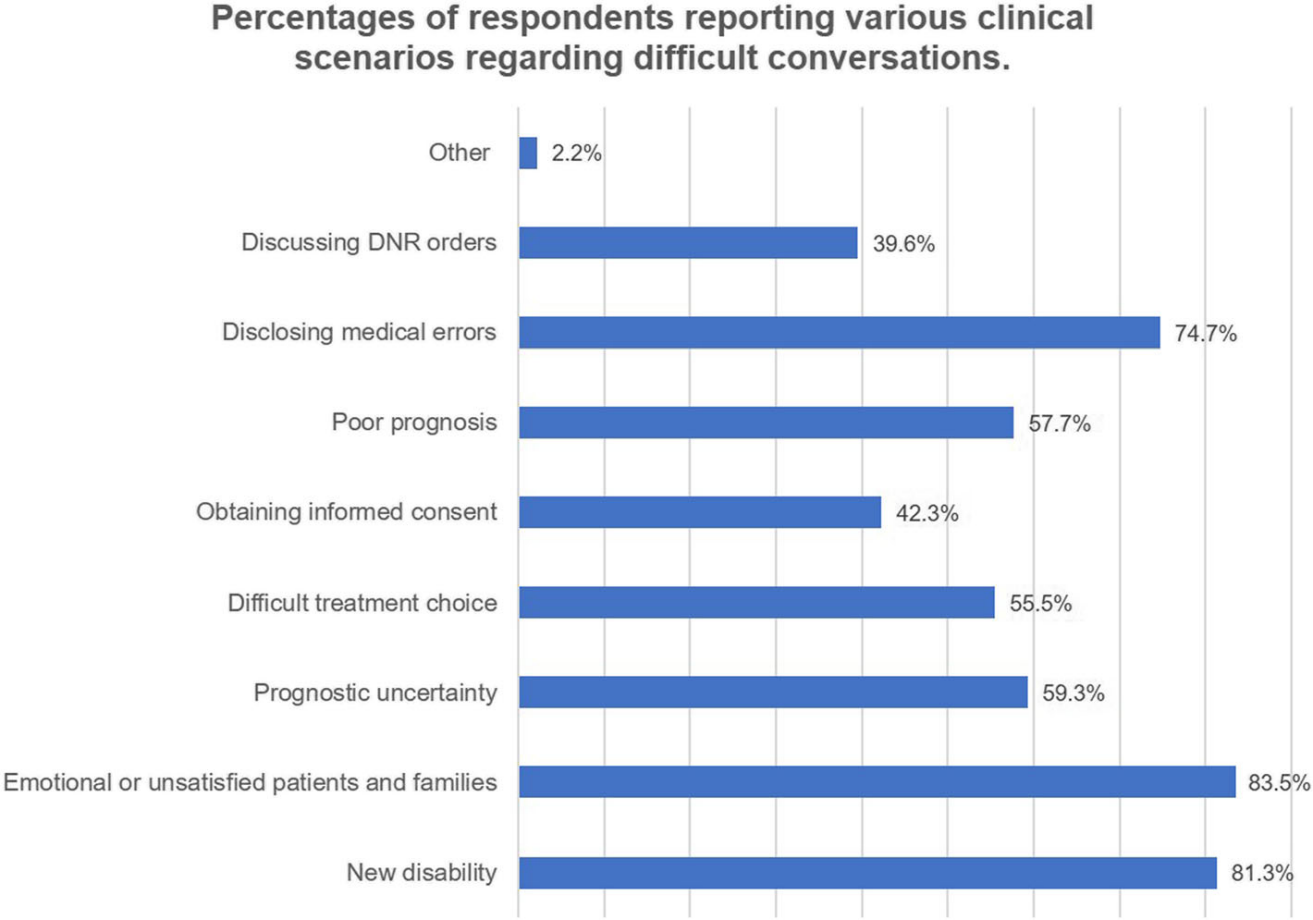
Percentages of respondents reporting various clinical scenarios regarding difficult conversations. Four respondents reported “other clinical scenarios”, such as urging patients to pay hospital fees

Residents reported a variety of clinical scenarios regarding difficult conversations they had experienced (Fig. 1). The most common scenario (*n* = 152, 83.52%) was dealing with emotional or unsatisfied patients and their families.

**Fig. 2:**
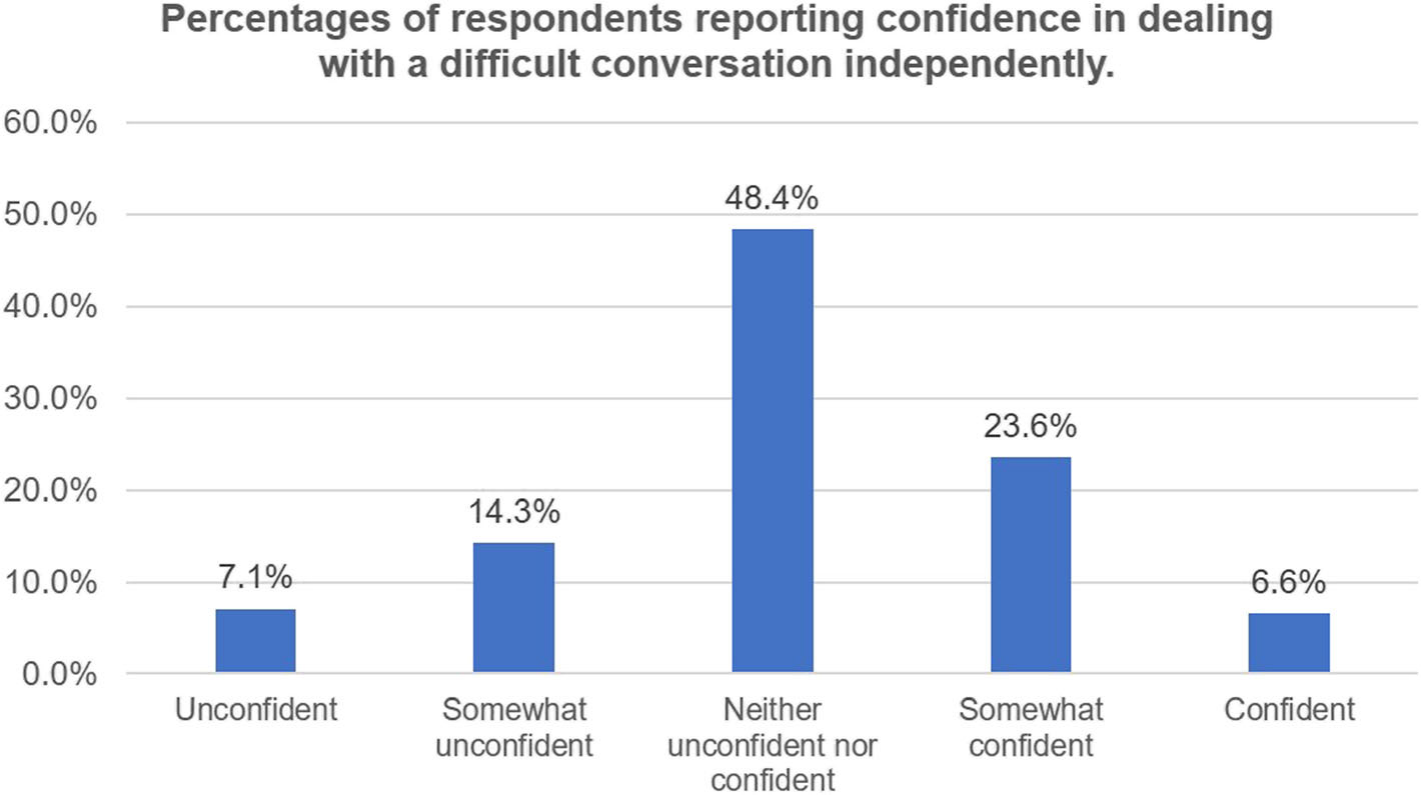
Percentages of respondents reporting confidence in dealing with a difficult conversation independently

**Fig. 3:**
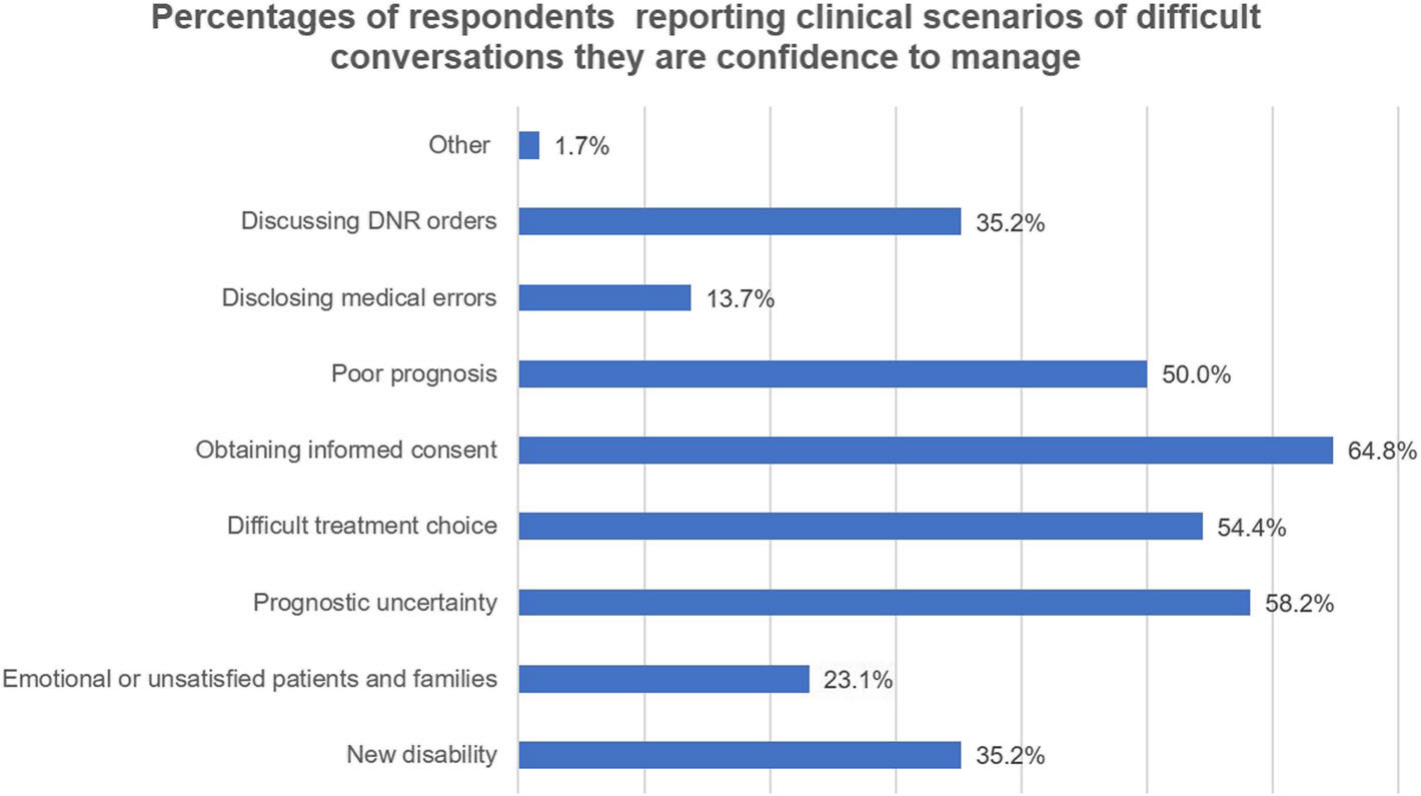
Percentages of respondents reporting clinical scenarios of difficult conversations they are confidence to manage. There were 3 residences reported “other” answers. But they did not describe the answer precisely

**Fig. 4:**
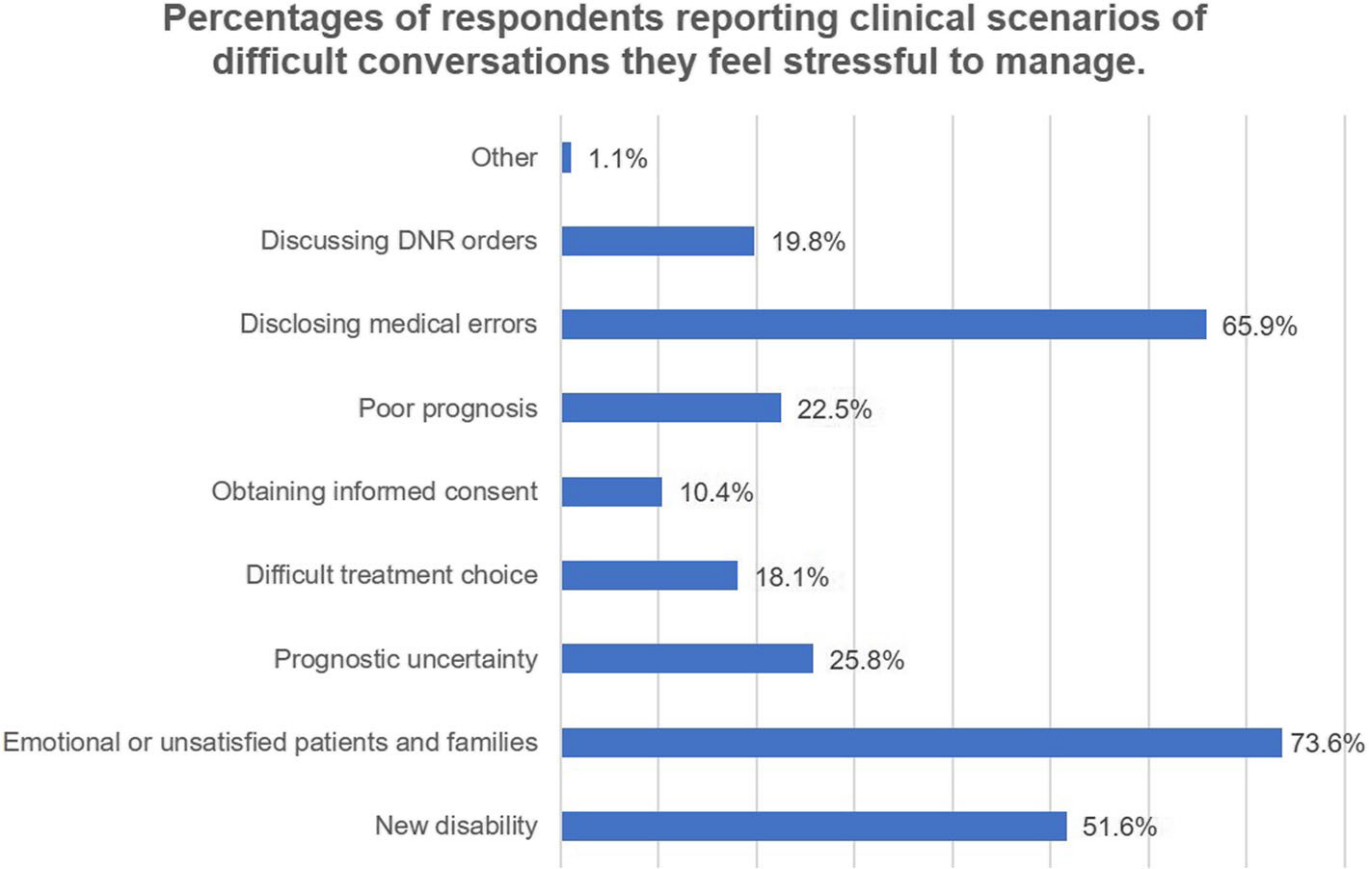
Percentages of respondents reporting clinical scenarios of difficult conversations they feel stressful to manage. There were 2 residences reported “other” answers including noisy environment and urging patients to pay hospital fees

The majority of respondent residents (*n* = 168; 92.31%) reported being exposed to at least one difficult conversation in their medical careers. One hundred forty-two residents (78.02%) had independently led a difficult conversation, of whom 82.39% (*n* = 117) reported having failed experiences in difficult conversations. Only 30.22% residents (*n* = 55) indicated being confident in independently dealing with a difficult conversation (Fig. 2). The most common clinical scenario of a difficult conversation that the residents reported having confidence managing was obtaining informed consent (*n* = 116, 58.24%) (Fig. 3). The most common clinical scenarios of difficult conversations that the residents reported feeling heavy pressure to manage were dealing with emotional or unsatisfied patients and their families (*n* = 134, 73.63%) and disclosing medical errors (*n* = 120, 65.93%) (Fig. 4).

### Previous formal training in communication skills during neurology residency and the correlation with confidence in managing difficult conversations

**Fig. 5:**
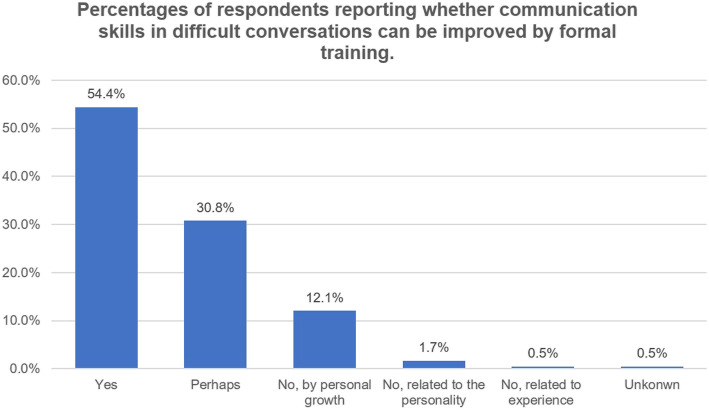
Percentages of respondents reporting whether communication skills in difficult conversations can be improved by formal training

The majority of respondent residents (*n* = 162, 89.01%) demonstrated that efficient management of difficult conversations is a vital part of clinical procedures. However, only 99 (54.4%) believed that communication skills in difficult conversation can be improved through formal training (Fig. 5).

Among 182 respondent residents, only 43 (23.63%) reported having previously received formal communication skills training. In comparison with residents without previous training, those with previous training indicated significantly more confidence and were under lower pressure in managing difficult conversations (*P* < 0.05) (Table 2).

**Table 2:** Comparison between residents with prior training and without prior training

	Residents receiving previous training (*n* = 43)	Residents not receiving previous training (*n* = 139)	P
Female (%)	35 (81.4%)	119 (85.6%)	0.504
Postgraduate level			0.024
PGY1	11 (25.6%)	54 (38.8%)	
PGY2	11 (25.6%)	44 (31.7%)	
PGY3	15 (34.9%)	30 (21.6%)	
PGY4	1 (2.3%)	5 (3.6%)	
PGY5	5 (11.6%)	6 (4.3%)	
Confidence in managing difficult conversations (1 means not confident)	3.47	2.96	0.003
Pressure in dealing with difficult conversation (1 means no pressure)	2.60	2.99	0.037

### Interest in receiving formal training in difficult conversations and barriers to implementation

**Fig. 6:**
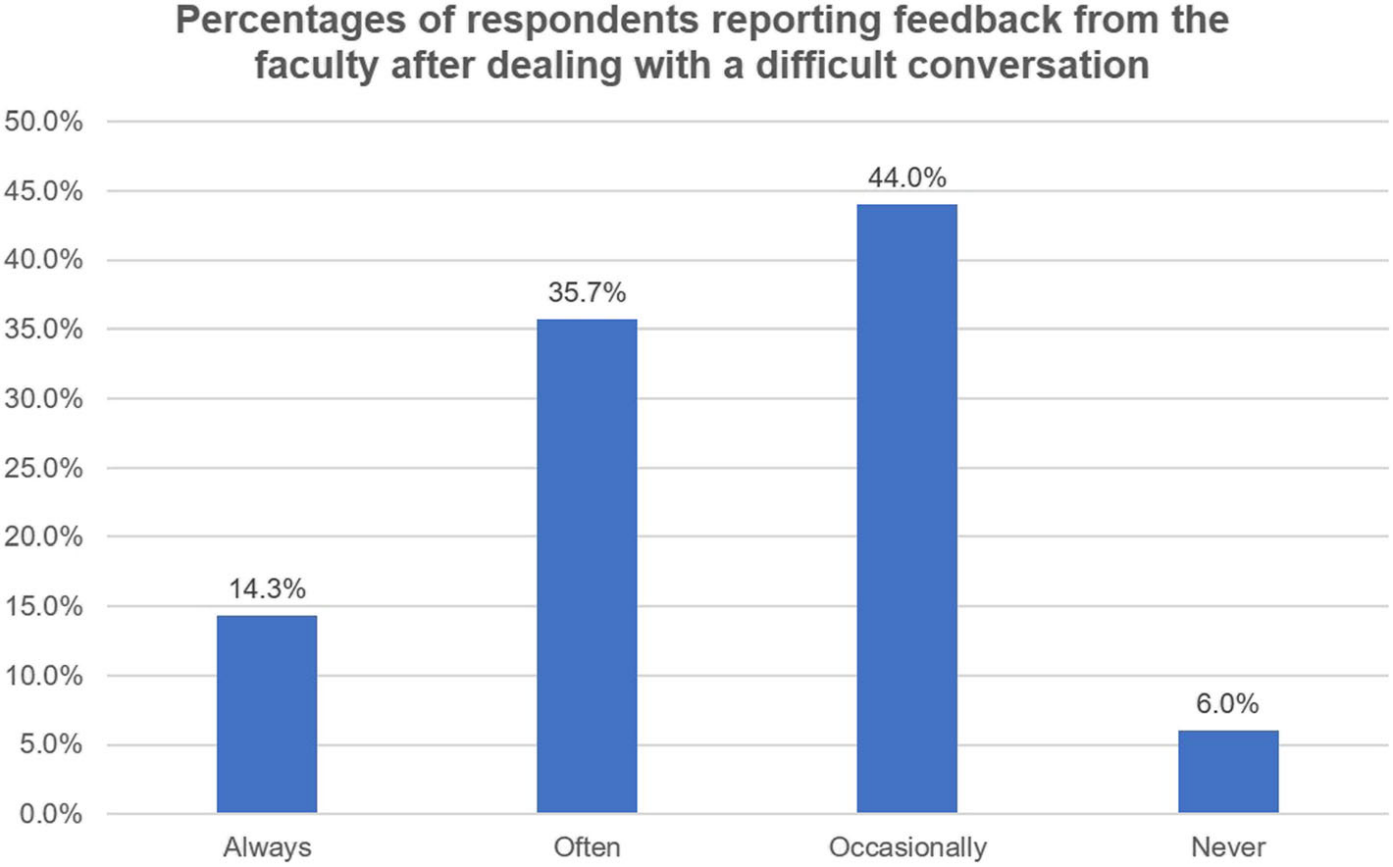
Percentages of respondents reporting feedback from the faculty after dealing with a difficult conversation

Most of the respondent residents (*n* = 165, 90.66%) believed that receiving formal training in difficult conversations was important for their careers. However, only 97 (53.3%) indicated interest in receiving formal training. Time, lack of enthusiasm, and lack of educational materials were the most commonly cited barriers to formalized training. Another important barrier was faculty expertise. Only 112 (65.74%) residents reported having experiences of being invited to participate in or observe difficult conversations between the faculty and patients or families. However, other residents (*n* = 70, 38.46%) expressed regret that they had been excluded from difficult conversations by the faculty. After managing difficult conversations, few residents (*n* = 26, 14.29%) believed they could get feedback from the faculty most of the time (Fig. 6).

## Discussion

For neurological residents, communication skill is a core competency of training due to the complexity and incurability of nervous system diseases [1, 9]. Work in the neurology discipline frequently involves the delivery of complex and difficult information to patients and their families, and effective communication is vital to the care of patients with neurological disease. Despite the importance of communication skills for neurologists, specific training in this area at the residency level is often lacking, especially regarding difficult conversations. Therefore, we sought to perform a needs assessment of difficult conversation education within neurology residency programs in five Chinese neurology training programs. To our knowledge, this is the first study to comprehensively assess the current situation of difficult conversation training in China. Overall, the results of this survey indicate that there is an unmet need for difficult conversation training in the participated neurology residency programs. This is actually not an unexpected finding, but it provides evidence for the need for further efforts to design and implement specific curriculum on this topic.

Our study showed that less than one quarter of residents had received prior training in communication skills, and the majority did not have enough confidence to independently manage difficult conversations. These results demonstrate that communication skills training in Chinese medical schools and post-graduate education might be insufficient. Moreover, most respondent residents reported great interest in receiving training in holding such difficult conversations. Therefore, our results suggest that there is a need to further develop and implement educational activities to teach residents to lead difficult conversations.

Our results indicated that difficult conversations are quite regular and common in neurological clinical care and almost all residents would be exposed to different types of difficult conversations during their training periods. Various clinical scenarios were reported by respondent residents. These difficult conversations range from delivering a diagnosis to sensitive conversations about a new disability, palliative care, advanced care planning, or brain death, and many of these conversations occur within the context of substantial prognostic uncertainty. Our results demonstrated that the top three common scenarios in difficult conversations reported by Chinese residents were working with emotional or unsatisfied patients and their families, disclosing bad news, and disclosing medical errors, which might be different from findings from other countries. As is well known, the physician-patient relationship in China is highly strained [11]. Therefore, managing difficult patients and their families might be the most stressful aspect of work for our clinicians, especially for inexperienced residents. Our results identified specific target areas in difficult conversations for neurology residency programs in China and provide evidence for developing future targeted curriculum in difficult conversations.

Communication skills training curriculum has been introduced into China for more than 10 years, and have been increasingly described in some other medical disciplines, including surgery and oncology recently [4, 12]. These studies have demonstrated that communication skills training program can improve the communication competency of residents. Our study showed that residents who received prior communication skills training had more confidence and faced less stress when they encountered difficult conversations. Although such a comparison does not represent that the previous training can improve the communication skills of the residents, this positive emotion may make them more enthusiastic to participate in practice of difficult conversations, and also encourage them to participate in future training. A few studies in neurological communication skills training have offered some evidence-based templates for curriculum development [10, 13]. The next step is to develop and implement a formal target curriculum in difficult conversations for our neurology residency programs. At the end of training, residents should be prepared to effectively communicate complex and difficult information to patients and families.

Our results noted barriers in developing and implement difficult conversation training including emotions, fears, time constraints, and a lack of opportunity; these are similar to results have been described previously in other medical and surgical specialties [14]. Another important barrier was the expertise of the teaching faculty. Our residents were often excluded when they encountered difficult conversations in clinical practice that were being held by faculty, which led them lose opportunities for observing and learning. Therefore, it is important to consider the development of communication curricula in residency with the goal of equipping the teaching faculty with skills to effectively teach and assess these skills. Efforts should be made to ensure that residents have opportunities to observe and be involved in complex communication encounters throughout their clinical training.

One major limitation in our study is the representativeness of the data. As we known, the scale of the training programs and the number of residencies is huge and the level of training quality is uneven in China. Therefore, it is quite difficult to conduct a survey including all training programs in China. Although the five programs were not randomly selected from all programs qualified to provide neurology residency training and cannot represent the overall situation in China, the main reason why we choose these five programs is that they are all from grade-A, class-3 teaching hospitals and the first batch of training programs to carry out standardized residency training in neurology in China. They represent the highest level of neurology residency training. Furthermore, the five hospitals are located in the North, West, East and Middle of China. Each hospital receives trainees from the surrounding hospitals at all levels, which is representative of the geographical distribution. Therefore, such survey can reflect the situation of residency training in neurology in China to a certain extent.

Another limitation is that our survey included only residents. Program directors, patients and their families were not included, which might result in reporting bias. Program directors have unique perspectives on their program’s successes and weaknesses. Future studies including program directors’ evaluation and patients and their family members’ evaluation will be necessary for curriculum development.

## Conclusion

This survey provides a preliminary assessment of the current status of education on the topic of difficult conversations in five accredit Chinese neurology residency training programs. Our results suggest that there is an unmet need to further develop and implement educational activities by teaching residents to lead difficult conversations. Future efforts should be made to establish and promote targeted communication curriculum in difficult conversations for Chinese neurological residents.

## Supplementary information

**Additional file 1.**

## Data Availability

The datasets used and/or analyzed during the current study are available from the corresponding author on reasonable request.

## Abbreviations

ACGME: Accreditation Council for Graduate Medical Education
PGY: Postgraduate year
PUMC: Peking Union Medical College
CAMS: Chinese Academy of Medical Science
PUMCH: Peking Union Medical College Hospital
