# Supplementary material for: Needs assessment for a curriculum for difficult conversations -a survey from 5 Chinese accredited neurology residency training programs
Source: BMC Med Educ. 2020 Sep 29;20:336. doi: 10.1186/s12909-020-02246-7 (PMC7525953; doi:10.1186/s12909-020-02246-7)
Supplement: Supplementary file 1 — Additional file 1. [file 12909_2020_2246_MOESM1_ESM.docx]

Needs assessment questionnaire for difficult conversations in a neurology residency training program

This questionnaire is designed to evaluate the status of neurology residency training in communication skills. We guarantee that all the collected information will be used for research purposes only. Your personal information will be kept confidential. Thank you very much for taking time off your busy schedule to complete our questionnaire! We hope that our questionnaire will be able to strive for more benefits for residents!

If you have any question, please feel free to talk to our staff distributing the questionnaire!

1. Seniority:

1. PGY 1; 2.PGY2; 3. PGY3; 4. PGY4; 5.PGY5 and more

1. Gender:
2. Male; 2.Female.
3. Age: __________ years old.
4. The highest education level obtained:
5. Bachelor’s degree; 2. Master’s degree; 3. Doctoral degree. 4. Postdoctoral degree
6. As your opinion, which areas should residents receive in the neurology residency training program?
7. Knowledge in Neurology;
8. Training in clinical reasoning
9. Communication skill training
10. Training in clinical research
11. Practice-based learning and improvement
12. Other:
13. As your opinion, which areas have been conducted well in the current neurology residency training program?
14. Knowledge in Neurology;
15. Training in clinical reasoning
16. Communication skill training
17. Training in clinical research
18. Practice-based learning and improvement
19. Other:
20. As your opinion, which areas have been neglected in the current neurology residency training program?
21. Knowledge in Neurology;
22. Training in clinical reasoning
23. Communication skill training
24. Training in clinical research
25. Clinical competence training in practice
26. Other:
27. Please declare the clinical scenarios regarding difficult conversations you have experienced:
28. Breaking bad news of new disability
29. Communication with emotional or unsatisfied patients and their families
30. Breaking bad news of prognostic uncertainty
31. Obtaining important inform consent (such as brain biopsy)
32. Breaking bad news of poor prognosis
33. Disclosing medical errors
34. Discussing DNR orders
35. Other:
36. Have you ever experienced difficult conversation?
37. Yes
38. No
39. Have you ever led a difficult conversation independently?
40. Yes
41. No
42. Did you have a failed experience, when you led a difficult conversation?
43. Yes
44. No
45. Did you feel confident when you led a difficult conversation independently?

0~5, 0 means no confidence, 5 means total confidence.

1. Which clinical scenarios regarding difficult conversations did you have confidence to manage?
2. Breaking bad news of new disability
3. Communication with emotional or unsatisfied patients and their families
4. Breaking bad news of prognostic uncertainty
5. Obtaining important inform consent (such as brain biopsy)
6. Breaking bad news of poor prognosis
7. Disclosing medical errors
8. Discussing DNR orders
9. Other:
10. Did you feel stressful when you led a difficult conversation independently?

0~5, 0 means not stressful at all, 5 means fully stressful.

1. Which clinical scenarios regarding difficult conversations did you feel more stressful to manage?
2. Breaking bad news of new disability
3. Communication with emotional or unsatisfied patients and their families
4. Breaking bad news of prognostic uncertainty
5. Obtaining important inform consent (such as brain biopsy)
6. Breaking bad news of poor prognosis
7. Disclosing medical errors
8. Discussing DNR orders
9. Other:
10. Did you receive a formal communication skill training previously?
11. Yes
12. No
13. Did you prepare before you lead a difficult conversation?
14. No
15. Yes, I will think about the content about the difficult conversation
16. Yes, I will make an outline about the difficult conversation
17. Yes, I will ask the faculty for the guidance about the difficult conversation
18. Do you feel the effective difficult conversation is important to the clinical procedure?

0~5, 0 means not important at all, 5 means absolutely important

1. Do you think communication with patients can be improved through training?
2. Yes
3. Perhaps
4. No, by personal growth
5. No, related to the personality
6. Do you think it is necessary for you to receive formal training in difficult conversations?
7. Yes
8. No
9. Would you like to receive formal training in difficult conversations?
10. Yes, very willing
11. Yes, but I do not have enough time
12. Yes, but I think bedside training is enough
13. No, I have no enthusiasm
14. When you faced a difficult conversation, would you like to lead it by yourself or let faculty to lead it?
15. Lead it by myself
16. Let faculty to lead it, and I observe
17. Lead it by myself, but I want faculty to observe the procedure
18. When the faculty led a difficult conversation, did you have willing to observe?
19. Yes
20. No
21. Did the faulty invite you to observe when he led a difficult conversation?
22. Yes
23. No
24. Was it helpful to you after you observed a difficult conversation led by the faculty?
25. Yes
26. No
27. Did you take the initiative to ask about the content and methods of the conversation to the faculty after your observation?
28. Yes
29. No
30. Did the faculty give you feedbacks after a difficult conversation?
31. Always
32. Often
33. Occasionally
34. Never
35. No, related to the experience
36. Unknown
